# CRISPR interference-guided multiplex repression of endogenous competing pathway genes for redirecting metabolic flux in *Escherichia coli*

**DOI:** 10.1186/s12934-017-0802-x

**Published:** 2017-11-03

**Authors:** Seong Keun Kim, Wonjae Seong, Gui Hwan Han, Dae-Hee Lee, Seung-Goo Lee

**Affiliations:** 1 Synthetic Biology and Bioengineering Research Center, Korea Research Institute of Bioscience and Biotechnology (KRIBB), Daejeon, 34141 Republic of Korea; 2 Department of Biosystems and Bioengineering, KRIBB School of Biotechnology, University of Science and Technology (UST), Daejeon, 34113 Republic of Korea

**Keywords:** CRISPR interference, *Escherichia coli*, *n*-Butanol, Multiple gene knockdown, Endogenous gene

## Abstract

**Background:**

Multiplex control of metabolic pathway genes is essential for maximizing product titers and conversion yields of fuels, chemicals, and pharmaceuticals in metabolic engineering. To achieve this goal, artificial transcriptional regulators, such as clustered regularly interspaced short palindromic repeats (CRISPR) interference (CRISPRi), have been developed to specifically repress genes of interest.

**Results:**

In this study, we deployed a tunable CRISPRi system for multiplex repression of competing pathway genes and, thus, directed carbon flux toward production of molecules of interest in *Escherichia coli*. The tunable CRISPRi system with an array of sgRNAs successfully repressed four endogenous genes (*pta*, *frdA*, *ldhA*, and *adhE*) individually and in double, triple, or quadruple combination that are involved in the formation of byproducts (acetate, succinate, lactate, and ethanol) and the consumption of NADH in *E. coli*. Single-target CRISPRi effectively reduced the amount of each byproduct and, interestingly, *pta* repression also decreased ethanol production (41%), whereas *ldhA* repression increased ethanol production (197%). CRISPRi-mediated multiplex repression of competing pathway genes also resulted in simultaneous reductions of acetate, succinate, lactate, and ethanol production in *E. coli*. Among 15 conditions repressing byproduct-formation genes, we chose the quadruple-target CRISPRi condition to produce *n*-butanol in *E. coli* as a case study. When heterologous *n*-butanol-pathway enzymes were introduced into *E. coli* simultaneously repressing the expression of the *pta*, *frdA*, *ldhA*, and *adhE* genes via CRISPRi, *n*-butanol yield and productivity increased up to 5.4- and 3.2-fold, respectively.

**Conclusions:**

We demonstrated the tunable CRISPRi system to be a robust platform for multiplex modulation of endogenous gene expression that can be used to enhance biosynthetic pathway productivity, with *n*-butanol as the test case. CRISPRi applications potentially enable the development of microbial “smart cell” factories capable of producing other industrially valuable products.

**Electronic supplementary material:**

The online version of this article (10.1186/s12934-017-0802-x) contains supplementary material, which is available to authorized users.

## Background

Modulation of gene expression is essential for balancing metabolic flux in metabolic pathway engineering, because balanced expression of heterologous pathway genes usually results in high productivity, product titer, and conversion yield [1–3]. The primary goal of balancing a metabolic pathway is to produce additional target products by reducing potential flux imbalances in the host cells. This is mainly achieved by eliminating the production of excessive intermediates or byproducts, which results in the efficient conversion of substrates, intermediates, and cofactors to desired products [4]. To prevent byproduct formation, host cells are typically engineered by knocking out genes involved in endogenous pathways associated with byproduct formation and that share the important intermediates and compete with heterologous production pathways [5]. However, consecutive deletion of multiple genes in competing pathways using the phage λ Red recombinase method widely used in *Escherichia coli* requires iterative recombination, which is irreversible, time consuming and laborious [6]. Furthermore, the phage λ Red recombinase method leaves unwanted scar-DNA sequences in host cells.

Multiplex automated genomic engineering (MAGE) and its derivatives have been developed and optimized to accelerate genome engineering by simultaneous modification of multiple genomic locations, including mismatches, insertions, and deletions [7]. However, MAGE exhibits limited applicability to diverse microbial hosts, because it requires a certain strain deficient in the DNA-mismatch-repair system, and the frequency of desired variants harboring multiple mutations is much lower than that of single-mutation variants [7, 8]. Recently, clustered regularly interspaced short palindromic repeats (CRISPR)-mediated genome engineering in conjunction with the λ Red recombinase method or MAGE was developed to rapidly manipulate multiple genes [9, 10] or integrate large DNA fragments into the *E. coli* chromosome [11, 12]. Besides simultaneous deletion of competing-pathway genes, repression of multiple genes can be considered an alternative approach for balancing the metabolic pathway. Repression of endogenous genes has been used for efficient production of desired metabolites in *E. coli* [13–16]. One benefit of gene repression is its ability to modulate endogenous gene expression without the modification of chromosomal DNA sequences. Furthermore, using the gene repression method, essential endogenous genes in host cells can be regulated [17], and the expression of target genes can be efficiently tuned to balance cell growth and the production of metabolites of interest [18, 19]. A general strategy for modulating gene expression at the translational stage using synthetic small-regulatory RNA (sRNA) was developed and successfully applied to metabolic engineering by combinatorial knockdown of endogenous and exogenous genes in *E. coli* [20]. Using the synthetic sRNA-based strategy, cadaverine titers in engineered *E. coli* increased by 55% under conditions of *murE* repression [13]. However, simultaneous expression of four synthetic sRNAs for repression of multiple genes imposes metabolic burden onto *E. coli* cells, because the efficiency of synthetic sRNA-based repression relies upon their binding affinity with target mRNA [13].

Recently, CRISPR interference (CRISPRi) was developed for DNA-sequence-specific gene regulation and used to repress multiple genes simultaneously in bacteria, yeast, plants, and animals [13, 21, 22]. CRISPRi enables the control of gene expression at the transcriptional level by blocking transcription initiation or elongation depending on single-guide RNA (sgRNA) binding sites [21]. CRISPRi implementation is simple and easy, because it requires only co-expression of a nuclease deficient Cas9 (dCas9) protein and an sgRNA that recognizes target gene sequences. As proof-of-concept applications to the metabolic engineering of *E. coli*, CRISPRi-mediated gene repression increased biosynthesis-pathway flux associated with the biodegradable material polyhydroxyalkanoate [14] and a plant flavonoid, naringenin [23]. Recently, we developed a regulatable CRISPRi system for fine-tuning biosynthetic pathways and, thus, directing carbon flux toward target-product synthesis. By exploiting engineered *E. coli* harboring a biosynthetic mevalonate (MVA) pathway and plant-derived terpenoid synthases, our bacterial CRISPRi system successfully modulated the expression of all MVA-pathway genes, resulting in enhanced production of isoprene, (−)-α-bisabolol, and lycopene [16]. However, most of these previous studies were focused on repression of heterologous pathway gene [16, 24] or single endogenous gene [25, 26] for enhanced production of molecules of interest. There remain only a handful of CRISPRi applications capable of simultaneous repression of multiple endogenous genes to promote enhanced production of target molecules [14, 23].

Acetyl-CoA is a key building block for the microbial production of fuels and chemicals [27, 28], such as *n*-butanol [29], polyhydroxybutyrate [30], and terpenoids [31, 32]. Therefore, substantial efforts have been undertaken to produce acetyl-CoA-driven molecules in microorganisms in order to improve acetyl-CoA availability by engineering pathways that consume or produce acetyl-CoA [27–31, 33]. Most efforts concerning acetyl-CoA-pool engineering in *E. coli* mainly focused on consecutive deletion of competing pathways, especially the acetate, lactate, and ethanol pathways [29, 33, 34]. However, this strategy is considered irreversible, time consuming and labor intensive, because several candidate strains need to be compared to identify the best-performing *E. coli* strain for production of molecules of interest from acetyl-CoA. Furthermore, metabolic models are increasingly used to computationally identify genomic interventions required for maximum production of a target molecule. In these cases, CRISPRi is ideally suited as an alternative to achieving the repression of multiple genes in various strains for metabolic engineering, because exploratory and model-guided repression of a set of endogenous genes and all gene combinations can be rapidly assessed in different hosts.

Here, we extended the feasibility of CRISPRi as a promising tool for multiplex repression of competing pathways. As a model system, multiplex CRISPRi was used to enrich acetyl-CoA pools in *E. coli* by reducing the formation of byproducts (acetate, succinate, lactate, and ethanol), thereby directing carbon flux toward improved production of *n*-butanol. Using a tunable CRISPRi system, we explored the effect of individual or combinatorial repression of multiple endogenous genes involved in the formation of acetate, lactate, succinate, and ethanol in central metabolic pathways on *n*-butanol production in *E. coli*. Furthermore, we compared the performance of several *E. coli* strains in terms of *n*-butanol production under conditions of simultaneous repression of multiple endogenous genes through the use of CRISPRi. Finally, we implemented static or dynamic knockdown of multiple endogenous genes simultaneously through use of CRISPRi, leading to increased production of *n*-butanol. This study represents a valuable example of CRISPRi-mediated repression of multiple endogenous genes for rapid evaluation of multiplex metabolic engineering interventions and can be used to develop *E. coli* as a well-organized cell factory for producing numerous acetyl-CoA-derived products.

## Results and discussion

### CRISPRi-mediated repression of multiple endogenous genes

**Fig. 1:**
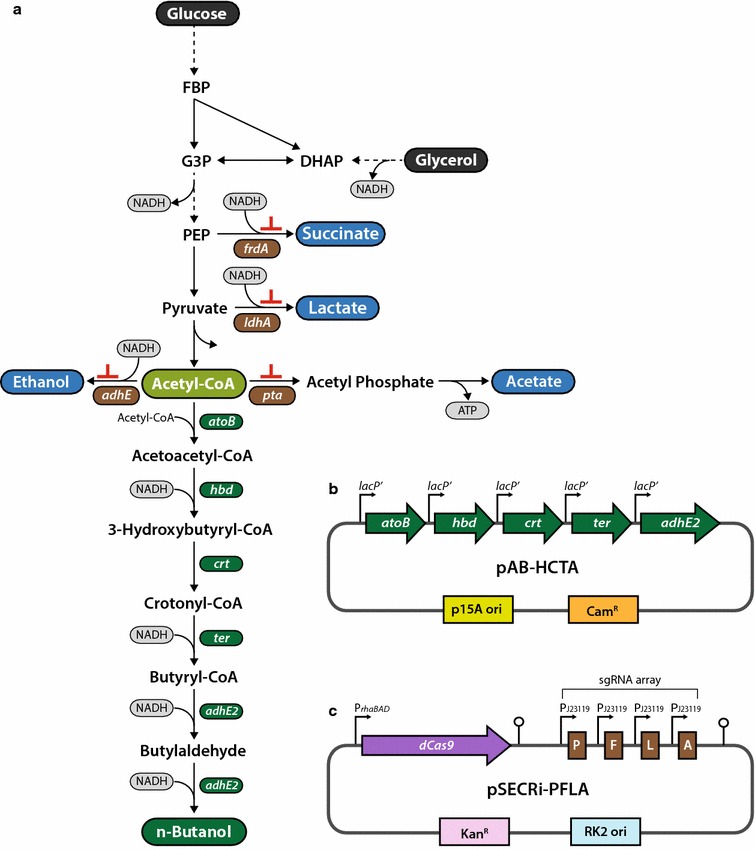
CRISPRi system design and construction for reducing byproduct formation in engineered *E. coli* producing *n*-butanol. **a** Schematic representation of the *n*-butanol-production pathway. The reconstituted *n*-butanol-production pathway consists of five enzymes involved in the six-step synthesis of *n*-butanol from acetyl-CoA. Glucose and glycerol mainly used for *n*-butanol production in *E. coli* are redirected into succinate, lactate, acetate, and ethanol production during glycolysis. **b** A plasmid encoding the five genes of the reconstructed *n*-butanol pathway. The expression of *n*-butanol-pathway genes was controlled by a constitutive *lac* promoter (*lacP′*). **c** The CRISPRi plasmid harboring an sgRNA array consisting of four sgRNAs targeting *pta*, *frdA*, *ldhA*, and *adhE* genes in *E. coli*. The CRISPRi system consisted of a dCas9 protein and sgRNAs governed by an l-rhamnose-inducible promoter (P_*rhaBAD*_) and a J23119 constitutive promoter (P_J23119_), respectively. P, F, L, and A are sgRNAs targeting endogenous *pta*, *frdA*, *ldhA*, and *adhE* gene, respectively. AdhE, aldehyde/alcohol dehydrogenase; AtoB, acetyl-CoA acetyltransferase; Cam^R^, chloramphenicol-resistance gene; Crt, crotonase; DHAP, dihydroxyacetone phosphate; FBP, fructose 1,6-bisphosphate; FrdA, fumarate reductase; G3P, glyceraldehyde 3-phosphate; Hbd, 3-hydroxybutyryl-CoA dehydrogenase; Kan^R^, kanamycin resistance gene; LdhA, lactate dehydrogenase; PEP, phosphoenolpyruvate; Pta, phosphate acetyltransferase; Ter, trans-enoyl-CoA reductase

To explore the feasibility of CRISPRi-mediated blockage of multiple competing pathways for redirecting carbon flux toward improved production of molecules of interest, we adopted the CRISPRi to control *n*-butanol-biosynthesis pathway flux as a model system. To this end, *E. coli* cells were transformed with a pAB-HCTA plasmid encoding a reconstituted *n*-butanol-production pathway that included five enzymes (AtoB, Hbd, Crt, Ter, and AdhE2) catalyzing six reactions from endogenous acetyl-CoA by incorporating their respective genes: *atoB* from *E. coli*, *hbd*, *crt*, and *adhE2* from *Clostridium acetobutylicum*, and *ter* from *Treponema denticola* (Fig. 1a, b) [35]. This synthetic pathway requires two molecules of acetyl-CoA and four molecules of NADH for production of one molecule of *n*-butanol (Fig. 1a). Glucose and glycerol have been mainly used for *n*-butanol production in engineered *E. coli*, wherein they are converted to acetyl-CoA through glycolysis. However, glucose and glycerol are mainly redirected into succinate, lactate, acetate, and ethanol production during glycolysis, which reduces the availability of cellular acetyl-CoA and NADH required for *n*-butanol production (Fig. 1a). Therefore, endogenous acetyl-CoA enrichment is expected to favor improved production of *n*-butanol in *E. coli*. In this context, we employed the regulatable CRISPRi system with an array of sgRNAs to channel carbon flows toward acetyl-CoA by simultaneously repressing the expression of four endogenous genes (*pta*, *frdA*, *ldhA*, and *adhE*) involved in production of acetate, succinate, lactate, and ethanol, respectively (Fig. 1c). Additionally, multiplex repression of four competing-pathway genes might conserve NADH required for *n*-butanol production in engineered *E. coli* because except for Pta enzyme, other three enzymes utilize NADH for their catalytic reactions. The CRISPRi system used here contained an l-rhamnose-inducible dCas9 expression cassette and an sgRNA array transcribed by a constitutive J23119 promoter in a single plasmid (pSECRi-PFLA) (Fig. 1c). We designed this plasmid based on the low-copy number pSEVA series to minimize metabolic burden; moreover, this plasmid can be easily swapped with other antibiotics and replication origins from the standard European vector architecture (SEVA) 2.0 database for use in applications with other bacterial hosts [36]. However, it is important to note that the functionality of the l-rhamnose and J23119 promoters must be determined on a case-by-case basis in other bacteria. To tightly control dCas9 expression, we used an l-rhamnose-inducible promoter with RhaS and RhaR regulators; however, 1.8 kb of the RhaS and RhaR regulators in CRISPRi plasmids can be removed to reduce plasmid size without altering regulatory functionality in *E. coli* [37]. Isopropyl β-d-1-thiogalactopyranoside (IPTG)-inducible promoters have been widely used for the expression of heterologous genes in metabolic engineering efforts. Therefore, we chose the l-rhamnose-inducible and J23119 promoters to allow for orthogonal control of the transcription of the *S. pyogenes* dCas9 gene and sgRNA, respectively, in the CRISPRi system used here. Moreover, the l-rhamnose-inducible promoter is capable of homogenous and rheostatic transcriptional control of genes and shows undetectable background expression in the absence of l-rhamnose [37, 38]. Because the *n*-butanol-production plasmid was derived from pACBB-eGFP (p15A origin, chloramphenicol resistance), and each gene of the *n*-butanol pathway is transcribed from a constitutive *lac* promoter (*lacP′*), it is genetically compatible with the pSECRi-PFLA plasmid (RK2 origin, kanamycin resistance), allowing *n*-butanol production in the absence of IPTG, which is an expensive inducer.

### Design and assay of sgRNAs targeting endogenous genes

To block multiple competing pathways of the synthetic *n*-butanol pathway in *E. coli*, we chose four repression-target genes based on their ability to compete for acetyl-CoA availability with the enzymes responsible for *n*-butanol production or to compete with the enzyme involved in acetyl-CoA production. These genes included *pta*, *frdA*, *ldhA*, and *adhE* encoding phosphate acetyltransferase, fumarate reductase, lactate dehydrogenase, and alcohol/aldehyde dehydrogenase, respectively (Fig. 1a). We then designed each sgRNA for repression of *pta*, *frdA*, *ldhA*, or *adhE* based on the following criteria: (1) we identified a conserved DNA sequence between *E. coli* K and B strains for application of the CRISPRi system to the diverse laboratory *E. coli* strains; (2) the sgRNA targets a non-template DNA strand in the 5′-proximal DNA sequence of each coding DNA sequence (CDS) to guarantee repression efficiency [15]; and (3) the 10-bp seed-DNA sequence harboring an NGG-protospacer-adjacent motif (PAM) domain cannot match perfectly with the *E. coli* host genome to avoid off-target effects of the dCas9/sgRNA complex. All designed sgRNA sequences are listed in Additional file 1: Table S3.

**Fig. 2:**
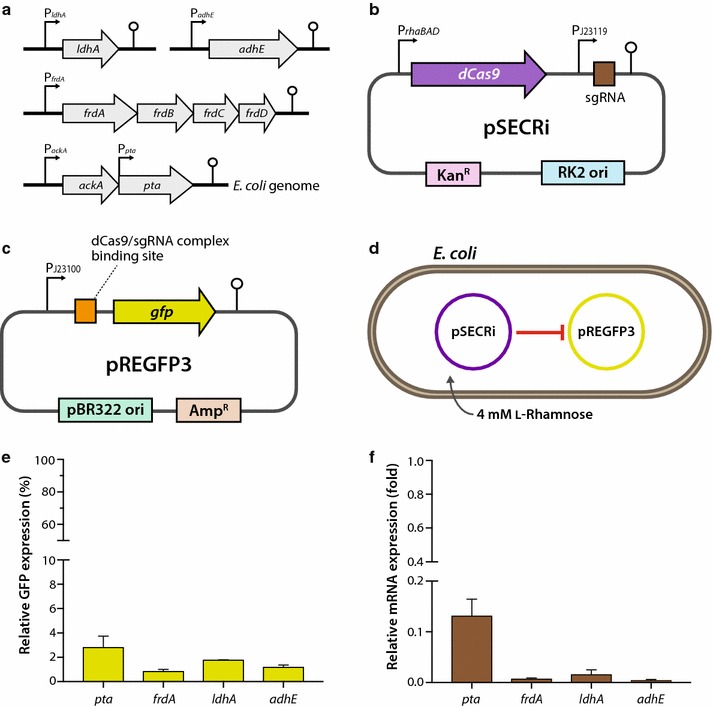
Schematic description of CRISPRi-mediated repression of endogenous genes involved in byproduct formation. **a** Genomic structure of four endogenous genes in *E. coli*. The *ldhA* and *adhE* genes are monocistronic, whereas *frdA* and *pta* reside in *frdABCD* and *ackA*-*pta* operon, respectively, in *E. coli*. In the *ackA*-*pta* operon, *pta* is controlled by a separate promoter in addition to the *ackA* promoter. **b** The CRISPRi plasmid harboring an sgRNA targeting the *pta*, *frdA*, *ldhA*, or *adhE* gene in *E. coli*. **c** A synthetic fluorescence-based gene-reporter plasmid containing a GFP-encoding gene. Binding sites for each dCas9-sgRNA complex were inserted between the constitutive J23100 promoter (P_J23100_) and the *gfp* gene in the pREGFP3 plasmid. The expression of the *gfp* gene was controlled by the constitutive J23100 promoter (P_J23100_). **d**, **e** In vivo fluorescence assay reporting CRISPRi-mediated GFP repression. Two plasmids (pSECRi and pREGFP3) were introduced into *E. coli*, and expression of the dCas9 protein was induced by 4 mM l-rhamnose. **f** mRNA levels of CRISPRi target genes in *E. coli*. The pSECRi plasmid was transformed into *E. coli* cells, and real-time PCR was performed to determine the mRNA levels of each gene (*pta*, *frdA*, *ldhA*, or *adhE*). The expression ratios of GFP were calculated as $expression\, (\% ) = \frac{{{{RFU_{xv} } \mathord{\left/ {\vphantom {{RFU_{xv} } {OD_{xv} }}} \right. \kern-0pt} {OD_{xv} }}}}{{{{RFU_{null} } \mathord{\left/ {\vphantom {{RFU_{null} } {OD_{null} }}} \right. \kern-0pt} {OD_{null} }}}}\; \times \;100,$ where RFU and OD are relative fluorescence units and optical density values at 600 nm, respectively. The subscript *xv* designates the tested cells harboring the pSECRi plasmid in the presence of l-rhamnose, whereas *null* indicates a control with the same pSECRi plasmid in the absence of l-rhamnose

In *E. coli*, the *ldhA* and *adhE* genes are monocistronic, whereas *frdA* and *pta* are resident in operons (Fig. 2a). Because the *frdA* gene is the first gene in the *frdABCD* operon, CRISPRi targeting *frdA* might repress the transcription of downstream *frdB*, *frdC*, and *frdD* genes comprising the fumarate reductase complex [23]. This is a potential benefit of CRISPRi-mediated transcriptional repression over translational-silencing strategies, such as sRNA. Given that genes encoding related enzymes in metabolic pathways are often grouped into operons similar to *frdABCD* and are frequently transcribed as polycistronic mRNA due to the lack of intervening terminators, targeting the first gene of an operon or a single promoter could repress many or all of the important enzymes in a metabolic pathway. Conversely, one disadvantage of this approach is that genes downstream of the target will be repressed if there is no intervening promoter, which could be problematic when downstream genes are essential, though unrelated to the pathway of interest, or required for any other reason [17]. Although *pta* is downstream of the *ackA* gene in the *ackA*-*pta* operon, the *pta* gene has its own promoter in addition to that of the *ackA* promoter [24]. Therefore, we targeted the *pta* promoter to block acetyl-CoA conversion to acetyl phosphate. After we chose the four repression-target genes and designed sgRNA sequences for *pta*, *frdA*, *ldhA*, and *adhE*, we performed a gene-reporter assay to assess the repression efficiency of the designed sgRNAs for CRISPRi. To this end, we used the pSECRi plasmid containing an l-rhamnose-inducible dCas9 expression cassette and sgRNA transcribed by a constitutive J23119 promoter (Fig. 2b) and a pREGFP3-reporter plasmid containing a binding site for an individual sgRNA with an NGG PAM sequence (Fig. 2c). This directed CRISPRi activity to the strand running opposite to the direction of transcription at a site between the *gfp* gene and its strong constitutive J23100 promoter. The repression activity associated with CRISPRi induced with 4 mM l-rhamnose was, thus, quantified by the reduction in green fluorescent protein (GFP) fluorescence (Fig. 2d). The reporter assay revealed that all CRISPRi systems based on the pSECRi plasmid with four sgRNAs (*ptaA*, *frdA*, *ldhA*, and *adhE*) successfully repressed *gfp* expression; the final GFP-expression levels ranged from 0.8 to 2.8% those of control values (Fig. 2e). Real-time PCR was performed to corroborate this result, verifying that the CRISPRi system functioned at the mRNA level to repress endogenous gene expression in *E. coli* harboring the pSECRi plasmid alone (Fig. 2f). Therefore, these results demonstrated that the l-rhamnose-inducible CRISPRi system was successfully used in the context of metabolic engineering for repression of endogenous competing-pathway genes in *E. coli*.

### Effect of CRISPRi-mediated single or combinatorial gene repression on byproduct formation

**Fig. 3:**
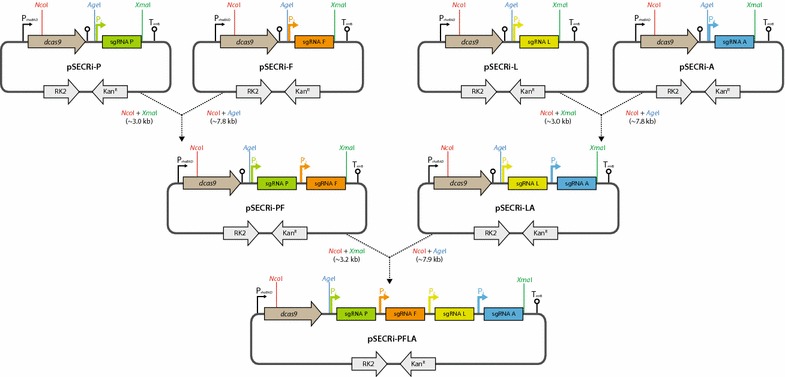
Construction of sgRNA arrays using a restriction digest-based modular assembly. Plasmids pSECRi-P, -F, -L, and -A encode individual sgRNAs that are flanked by *Age*I and *Xma*I on the 5′ and 3′ end, respectively. These flanking restriction sites in all pSECRi plasmids allow us to connect any two sgRNAs by ligating a plasmid backbone digested with *Nco*I and *Age*I to an insert fragment obtained by digestion with *Nco*I and *Xma*I. The resulting plasmid (pSECRi-PF, or -LA) harbors a two-sgRNA array that again is flanked by *Age*I and *Xma*I on the 5′ and 3′ end, respectively. We should mention that *Age*I and *Xma*I have compatible cohesive ends and ligation of both DNA fragments digested with *Age*I and *Xma*I generates a new restriction site that is not cleavable by both restriction enzymes. A two-sgRNA array can be joined to the array by ligating a plasmid backbone digested with *Nco*I and *Age*I to an insert digested with *Nco*I and *Xma*I, resulting in a pSECRi-PFLA. All sgRNAs are under the control of J23119 promoter (P_1_–P_4_)

After examination of individual repression efficiency by the designed sgRNAs using the fluorescence-reporter plasmid and real-time PCR, we created sgRNA arrays by assembling four individual sgRNAs (*pta*, *frdA*, *ldhA*, and *adhE*) into six double sgRNA arrays (*pta*/*frdA*, *pta*/*ldhA*, *pta*/*adhE*, *frdA*/*ldhA*, *frdA*/*adhE*, and *ldhA*/*adhE*), four triple sgRNA arrays (*pta*/*frdA*/*ldhA*, *pta*/*frdA*/*adhE*, *pta*/*ldhA*/*adhE*, and *frdA*/*ldhA*/*adhE*), and one quadruple sgRNA array (*pta*/*frdA*/*ldhA*/*adhE*) to conduct multiplex repression of endogenous genes in *E. coli*. To exchange the four individual sgRNAs, only a single cloning step using simple PCR and ligation was required. This allowed easy assembly of the sgRNA array for multiplex repression by two consecutive cloning steps involving individual sgRNA cassettes using a BioBrick assembly method using *Age*I/*Xma*I isocaudomers (Fig. 3). To this end, we placed sgRNAs in tandem, with *Age*I and *Xma*I restriction enzyme sites inserted at both ends of the sgRNA cassette (Fig. 3). *Age*I and *Xma*I have compatible cohesive ends, and the ligation of both DNA fragments digested with *Age*I and *Xma*I generates a new restriction site not cleavable by either of the two restriction enzymes. We transformed a pSECRi plasmid encoding single, double, triple, or quadruple sgRNA(s) into *E. coli* DH5α cells and cultivated them in terrific broth TB-glucose medium supplemented with 4 mM l-rhamnose, which is required for inducing dCas9 expression in the pSECRi plasmid, under micro-aerobic conditions. After a 36 h cultivation, we measured the concentrations of acetate, succinate, lactate, and ethanol in the culture medium.

All single CRISPRi systems reduced the formation of the corresponding byproduct (Table 1). The CRISPRi targeting the *pta* gene for acetate reduction was the least effective based on acetate production being reduced by only 31% as compared with that observed in a control strain harboring a pSEVA221 plasmid. Therefore, we designed three additional sgRNAs targeting the *pta* gene [P2 targeting the ribosome-binding site (RBS), P3 and P4 targeting the CDS at different locations] and used these to repress the *pta* gene in *E. coli* DH5α cells. The acetate concentrations produced from single CRISPRi using four different sgRNAs did not result in significantly different results (Additional file 1: Figure S1), indicating that the repression activity might not be further improved due to the *pta* gene being transcribed from both *ackA* and *pta* promoters. Otherwise, during the 36 h cultivation, acetate might also be generated from alternative acetate-production pathways (*poxB*, pyruvate dehydrogenase) involving glucose in *E. coli* [39, 40]. Interestingly, along with acetate reduction, ethanol production also decreased to 41%, with this reduction reproducible using all four sgRNAs targeting the *pta* gene (Additional file 1: Figure S1). This suggested that ethanol reduction was caused not by off-target effects of CRISPRi, but by reduced expression of phosphate acetyltransferase encoded by the *pta* gene, which was consistent with a previous study reporting that a *E. coli* BW25113Δ*pta* mutant produced less ethanol [41, 42]. CRISPRi-mediated *ldhA* repression produced less lactate (16% relative to the control), whereas production of succinate and ethanol was elevated to 131 and 197%, respectively (Table 1), indicating that *ldhA* repression increased the availability of pyruvate and, subsequently, acetyl-CoA. In our culture conditions, lactate was the most highly produced byproduct (7.1 g/L) with other byproducts (acetate, succinate, and ethanol) generated at 2.78, 0.82, and 0.68 g/L, respectively, in the control strain harboring a pSEVA221 plasmid (Table 1). Because lactate was largely reduced to 1.15 g/L from 7.1 g/L through CRISPRi-mediated *ldhA* repression, this might have affected the formation of other byproducts, such as ethanol, which could potentially solve the NADH-regeneration problem. Furthermore, repression of *frdA* and *adhE* decreased succinate and ethanol production by 25 and 4%, respectively, without altering formation of other byproducts.

**Table 1:** Comparison of the effect of multiplex CRISPRi on byproduct production in *E. coli*

Repression genes					Product concentrations (g/L)				Product change (%)^a^			
Number	*pta*	*frdA*	*ldhA*	*adhE*	Acetate	Succinate	Lactate	Ethanol	Acetate	Succinate	Lactate	Ethanol
None					2.78	0.82	7.10	0.68	0	0	0	0
Single	∇				1.92	0.91	7.18	0.27	− 31	+ 10	+ 1	− 59
		∇			2.59	0.21	7.10	0.62	− 7	− 75	0	− 8
			∇		2.97	1.08	1.15	1.33	+ 7	+ 31	− 84	+ 97
				∇	2.58	0.74	6.90	0.03	− 7	− 11	− 3	− 96
Double	∇	∇			2.24	0.49	7.83	0.62	− 20	− 41	+ 10	− 8
	∇		∇		3.30	1.33	3.51	1.07	+ 18	+ 61	− 51	+ 59
	∇			∇	2.09	0.80	7.19	0.14	− 25	− 2	+ 1	− 80
		∇	∇		2.91	0.37	1.76	1.46	+ 5	− 55	− 75	+ 116
		∇		∇	2.66	0.36	7.42	0.08	− 4	− 57	+ 5	− 88
			∇	∇	3.42	0.80	1.39	0.21	+ 23	− 2	− 80	− 70
Triple	∇	∇	∇		3.58	0.73	4.37	1.43	+ 29	− 12	− 38	+ 113
	∇	∇		∇	2.13	0.52	7.67	0.14	− 24	− 37	+ 8	− 79
	∇		∇	∇	3.25	0.96	3.96	0.57	+ 17	+ 17	− 44	− 16
		∇	∇	∇	3.72	0.65	1.56	0.59	+ 34	− 21	− 78	− 13
Quadruple	∇	∇	∇	∇	3.14	0.71	4.57	0.58	+ 13	− 14	− 36	− 14

^a^The percentage reduction of target byproduct was calculated as $\left[ {\frac{{{\text{iProduct}}\;\left[ {{{\text{g}} \mathord{\left/ {\vphantom {{\text{g}} {\text{L}}}} \right. \kern-0pt} {\text{L}}}} \right]}}{{{\text{Product}}\;\left[ {{{\text{g}} \mathord{\left/ {\vphantom {{\text{g}} {\text{L}}}} \right. \kern-0pt} {\text{L}}}} \right]}}\; \times \;100} \right] - 100$ where iProduct [g/L] and Product [g/L] are concentration of each target byproduct produced in the presence and absence of CRISPRi control, respectively

Using the double CRISPRi systems enabled repression of two target genes simultaneously (*pta*/*frdA*, *pta*/*ldhA*, *pta*/*adhE*, *frdA*/*ldhA*, *frdA*/*adhE*, *ldhA*/*adhE*). Similar to single CRISPRi, double CRISPRi also reduced byproduct production in all tested cases (Table 1). Double CRISPRi repressing *pta*/*ldhA* or *frdA*/*ldhA* genes decreased lactate production and increased ethanol production simultaneously, whereas the enhanced ethanol production was suppressed by double CRISPRi targeting *ldhA*/*adhE* genes (Table 1). The repression efficiencies of triple and quadruple CRISPRi were similar to that of double CRISPRi (Table 1). Notably, we observed that increasing the number of sgRNAs to repress endogenous genes involved in succinate, lactate, and ethanol biosynthesis and NADH consumption might cause an NAD^+^/NADH imbalance in host cells, because the FrdA, LdhA, and AdhE enzymes require NADH for catalysis in the central glycolytic pathway (Fig. 1a). Therefore, our findings showed that byproduct formation was severely influenced by other metabolic fluxes, especially during the process of multiple-gene repression. Overall, these results indicated that the multiplex CRISPRi system successfully repressed endogenous genes in *E. coli* and reduced byproduct formation. Additionally, we observed that ethanol production was dependent upon the expression of the *ldhA* gene, the product of which catalyzes the conversion of pyruvate to lactate in *E. coli*.

### Effects of CRISPRi-mediated single or combinatorial gene repression on *n*-butanol production

**Fig. 4:**
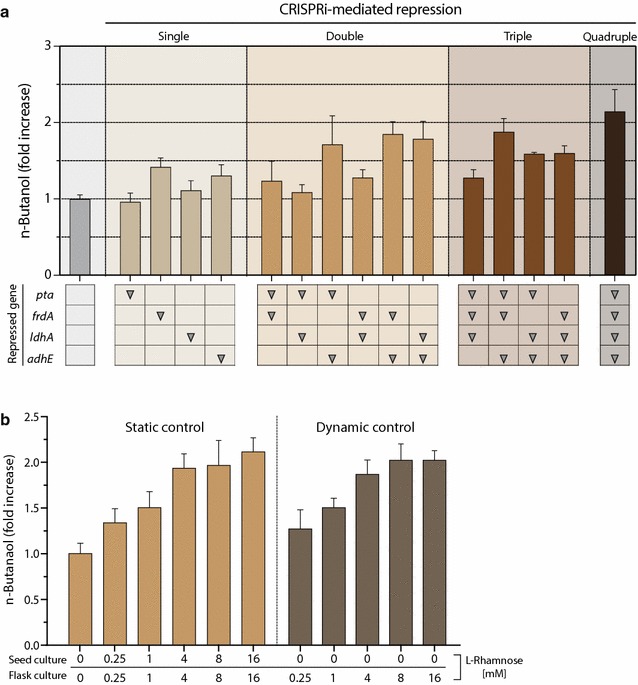
Comparison of the effect of CRISPRi-mediated multiplex gene repression on *n*-butanol production in *E. coli*. DH5α cells harboring pAB-HCTA and each pSECRi plasmid were grown in TB medium supplemented with 20 g/L glycerol and **a** 4 mM l-rhamnose or **b** various concentrations of l-rhamnose at 37 °C for 48 h. *n*-Butanol levels were determined by GC. The fold increase of *n*-butanol production was calculated as ${n{\text{-}}butanol}\; (fold) = \frac{{BtOH_{xv} }}{{BtOH_{null} }}\; \times \;100,$ where BtOH is *n*-butanol concentration. The subscript *xv* designates the tested cells harboring the pAB-HCTA and pSECRi plasmid in the presence of l-rhamnose, whereas *null* indicates a control with the same two plasmids in the absence of l-rhamnose. Data represent the averages of three biological cultures, and error bars show the standard deviation (SD)

Given the success of CRISPRi to repress multiple endogenous genes, resulting in byproduct reduction in *E. coli*, we examined whether *n*-butanol production is enhanced by CRISPRi-guided reductions in byproduct formation. Based on an our previous study [35], we transformed the pAB-HCTA plasmid encoding heterologous *n*-butanol-pathway genes into *E. coli* DH5α cells harboring the pSECRi plasmid encoding single or multiple sgRNA(s). Cells were grown in TB-glycerol medium supplemented with 4 mM l-rhamnose under micro-aerobic conditions, and accumulated *n*-butanol in the headspace of the culture bottle was analyzed after a 48 h cultivation. In the case of single CRISPRi, repression of *frdA* or *adhE* improved *n*-butanol production by up to 1.3- and 1.2-fold, respectively, whereas repression of *pta* or *ldhA* showed no significant effects on *n*-butanol production in *E. coli* (Fig. 4a). Multiplex repression of CRISPRi also increased *n*-butanol production, except for double CRISPRi targeting the *pta* and *ldhA* genes. Among the multiplex-repression systems, quadruple CRISPRi repressing all four genes (*pta*, *frdA*, *ldhA*, and *adhE*) resulted in the highest increase (2.1-fold) in *n*-butanol production in *E. coli*. To probe whether this improvement in *n*-butanol production was caused by CRISPRi-mediated multiplex repression, we used various concentrations of l-rhamnose ranging from 0 to 16 mM to induce dCas9 expression. We adopted two strategies, static and dynamic, to control dCas9 expression. For static regulation of dCas9 expression, we used the same concentrations of l-rhamnose in both seed and main cultures. For dynamic control, we did not add l-rhamnose to the culture media of the seed culture, and after transferring the seed culture to the main culture, we then added different concentrations of l-rhamnose (0.25, 1, 4, 8 or 16 mM). In both cases, higher concentrations of l-rhamnose (0.25–4 mM) resulted in higher production of *n*-butanol (Fig. 4b). However, the *n*-butanol-enhancing effect reached a plateau at 4 mM, i.e., *n*-butanol production slightly increased at 8 or 16 mM l-rhamnose. Based on these results, we switched the origin of replication in the pSECRi plasmid from RK2 (low copy number) to pBBR1 (medium copy number) to increase the production of the dCas9-sgRNA complex. However, we observed no subsequent improvement in *n*-butanol production in *E. coli* (Additional file 1: Figure S2), which suggested that the ratio of dCas9 and sgRNA content may be a critical factor, i.e., trace amount of dCas9 is sufficient to repress a few copies of endogenous genes. In general, factors that determine CRISPRi efficiency are length, sequence complementarity, and binding location of sgRNA [22]. Because the dynamic control strategy required no l-rhamnose during seed cultivation, this approach would be more suitable for the development of economic bioprocesses for *n*-butanol production via CRISPRi.

### CRISPRi-mediated screening of the best *n*-butanol producing *E. coli*

**Fig. 5:**
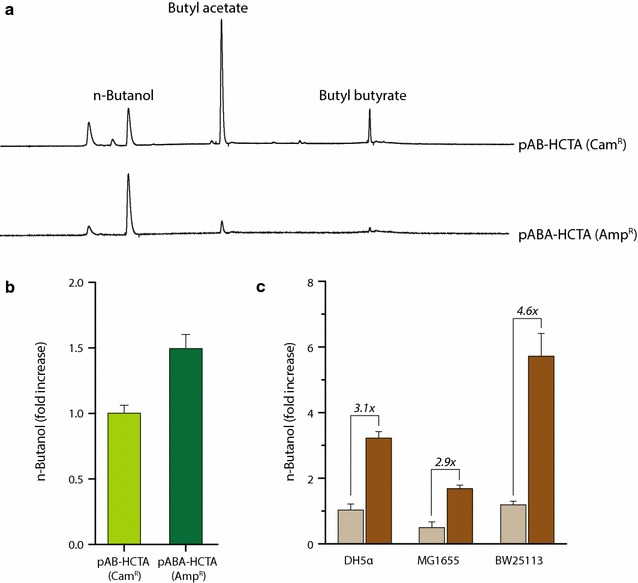
Reductions in *n*-butanol-derived byproducts and screening of various *E. coli* strains for *n*-butanol production. *Escherichia coli* DH5α cells containing pAB-HCTA(Cam^R^) or pABA-HCTA(Amp^R^) were grown in TB medium supplemented with 20 g/L glycerol at 37 °C for 48 h. **a**, **b** Metabolic products, including *n*-butanol and its derivatives, were determined by GC. The fold increase of *n*-butanol production was calculated as $n{\text{-}}butanol\; (fold) = \frac{{BtOH_{amp} }}{{BtOH_{cam} }} \times 100,$ where BtOH is *n*-butanol concentration. The subscript *amp* designates the tested cells harboring the pABA-HCTA, whereas *cam* indicates a control with the pAB-HCTA plasmid. **c** To screen the best *n*-butanol producer among various *E. coli* strains, three different *E. coli* strains (DH5α, MG1655, and BW25113) were examined. Two plasmids, pSECRi-PFLA carrying a quadruple sgRNA array and pABA-HCTA encoding heterologous *n*-butanol-pathway genes, were introduced into each *E. coli* strain, which was grown in TB medium supplemented with 20 g/L glycerol and 4 mM l-rhamnose at 37 °C for 48 h. *n*-Butanol levels were determined by GC. The fold increase of *n*-butanol production was calculated as $n{\text{-}}butanol \;(fold) = \frac{{BtOH_{xv} }}{{BtOH_{dh} }}\; \times \;100,$ where BtOH is *n*-butanol concentration. The subscript *xv* designates the tested cells harboring the pABA-HCTA and pSECRi-PFLA, whereas *dh* indicates *E. coli* DH5α cells with the pABA-HCTA and pSEVA221. Data represent the averages of three biological cultures, and error bars show the standard deviation (SD)

During analysis of *n*-butanol and byproduct production, butyl acetate and butyl butyrate were unexpectedly detected in *E. coli* cells harboring pAB-HCTA (Fig. 5a). A recent study showed that the chloramphenicol-resistance gene (*cat*) encoding chloramphenicol acetyltransferase (CAT) attaches an acetyl or butyryl group derived from acetyl-CoA or butyryl-CoA, respectively, to one of the hydroxyl groups on the aromatic alcohols [1], butanol and methyl butanol [43]. Coincidentally, because the pAB-HCTA plasmid encoding heterologous *n*-butanol-pathway genes contained the *cat* gene, we hypothesized that butyl acetate and butyl butyrate were produced from *n*-butanol acetylation by the CAT enzyme. To test this, we replaced the *cat* gene with an ampicillin-resistance gene (β-lactamase encoded by the *bla* gene) in the pAB-HCTA plasmid. As expected, *E. coli* cells harboring an *n*-butanol-producing plasmid with ampicillin resistance produced almost no butyl butyrate, butyl acetate formation also significantly reduced (Fig. 5a). Additionally, *n*-butanol production increased 1.5-fold as compared with that observed in cells harboring the pAB-HCTA plasmid with the *cat* gene (Fig. 5b), indicating that CAT was involved in the production of butyl acetate and butyl butyrate. Therefore, we used the pAB-HCTA plasmid with ampicillin resistance (pABA-HCTA) for further experiments on *n*-butanol production. To screen for the best *n*-butanol producer among various *E. coli* strains, we used three different *E. coli* strains: DH5α, MG1655, and BW25113. We transformed two plasmids, pSECRi-PFLA carrying a quadruple sgRNA array and pABA-HCTA encoding heterologous *n*-butanol-pathway genes, into each *E. coli* strain. Each *E. coli* strain harboring the pABA-HCTA plasmid and a pSEVA221 plasmid (no repression) was also used as a control. In the absence of CRISPRi regulation, the BW25113 strain produced ~ 30% more *n*-butanol than the DH5α strain and 125% more *n*-butanol than the MG1655 strain (Fig. 5c). In the presence of CRISPRi regulation, *n*-butanol production increased in all tested *E. coli* strains relative to the control: 3.1-fold in the DH5α strain, 2.9-fold in the MG1655 strain, and 4.6-fold in the BW25113 strain. These results showed that the BW25113 strain exhibiting quadruple gene silencing produced 5.9-fold and 10.3-fold more *n*-butanol relative to the DH5α and MG1655 strain, respectively, in the absence of CRISPRi regulation. Therefore, we easily selected the BW25113 strain as the best *n*-butanol producer through use of CRISPRi-mediated multiple-gene repression. Indeed, the BW25113 strain has been mainly used as a host for *n*-butanol production in numerous studies [44, 45] since it was used in the pioneer work on *n*-butanol production [34]. In addition, a maximum titer of 30 g/L of *n*-butanol in *E. coli* was also achieved using BW25113 deletion mutants through anaerobic cultivation [33].

### Effects of multiplex CRISPRi on production of *n*-butanol

Glycerol may be an ideal feedstock to produce *n*-butanol because of its availability, low prices, and a high degree of reduction [46]. Therefore, to examine the effect of quadruple CRISPRi condition (*pta*, *frdA*, *ldhA*, and *adhE*) on production of *n*-butanol and byproducts from glycerol, instead of glucose, in *E. coli*, BW25113 cells containing the pSECRi-PFLA and pABA-HCTA plasmids were grown in TB medium supplemented with 20 g/L glycerol and 4 mM l-rhamnose for dCas9 expression under micro-aerobic batch fermentation, and concentrations of *n*-butanol and byproducts were monitored for 99 h. BW25113 strain containing a pSEVA221 plasmid instead of pSECRi-PFLA was also used as a control strain (Additional file 1: Figure S3 and Table 2). In the control strain, 20 g/L glycerol was completely consumed after 60 h, whereas there remained 8.21 g/L glycerol in the strain under CRISPRi regulation after 60 h. Additionally, production of ethanol and lactate was negligible in both strains, and acetate was continuously produced up to 2.14 g/L in the control strain, whereas strains under CRISPRi regulation yielded twofold less acetate (1.03 g/L). Succinate was the most highly produced byproduct in both strains, with the control strain producing 4.62 g/L succinate, and the strain under CRISPRi regulation produced 1.20 g/L succinate (Table 2). The *n*-butanol yield and productivity via CRISPRi regulation increased up to 5.4- and 3.2-fold, respectively. Overall, the strain under CRISPRi regulation successfully reduced succinate and acetate formation and increased *n*-butanol production from glycerol. The implementation of CRISPRi-mediated repression of multiple endogenous genes was simple and efficient for enhanced production of molecules of interest, and is applicable to nearly any other endogenous genes including essential genes for host cell growth.

**Table 2:** Effect of CRISPRi-mediated multiplex repression on *n*-butanol production

CRISPRi repression		
Analysis	Quadruple repression	No repression
Metabolite concentrations [g/L]		
Butanol	1.06	0.33
Acetate	1.03	2.14
Ethanol	0.0	0.0
Lactate	0.0	0.18
Succinate	1.20	4.62
Glycerol^a^	8.21	0.0
Cell growth [OD_600_]	3.96	5.48

Cells were grown micro-aerobically in TB-glycerol media at 37 °C for 60 h

^a^Glycerol remained

## Conclusions

Identification of potential target genes and balancing their expression are crucial for the efficient production of molecules of interests [47, 48]. However, it is limited to manipulate multiple target genes through the conventional approaches of gene deletion. This study described a simple method for multiple endogenous gene repression using a designed CRISPRi system. By simple inverse PCR, we created CRISPRi plasmids targeting four endogenous genes, resulting in the successful repression of GFP expression to 2.8% following transformation of multi-copy reporter plasmids and reductions in the product formation via endogenous target genes. Using BioBrick assembly, sgRNA arrays targeting two, three, or four genes were created within two successive cloning steps. Their subsequent utilization reduced byproduct formation simultaneously, although the effects of repression of one byproduct influenced the formation of other byproducts. When the four byproduct pathways were blocked by CRISPRi in the DH5α strain, *n*-butanol production was enhanced by up to 2.1-fold, with this enhancement dependent upon dCas9 expression. Furthermore, we found that the CAT enzyme responsible for conferring chloramphenicol resistance was involved in the formation of butyl acetate and butyl ester, and that by exchanging the *cat* gene for a *bla* gene and performing optimal-strain selection, *n*-butanol yield was enhanced by up to 5.9-fold as compared with that observed in the DH5α strain containing the production plasmid without CRISPRi regulation. When glycerol, instead of glucose, was used as the carbon source for *n*-butanol production under CRISPRi regulation, it also exhibited reduced formation of byproducts, especially succinate and acetate, resulting in higher *n*-butanol yields and productivity than those observed in unregulated strains. The CRISPRi system holds great promise as a genetic reprogramming platform that is suitable for microbial metabolic engineering. In particular, the ability of CRISPRi to effortlessly regulate multiple endogenous genes simultaneously will play a very important role for synthetic biology and metabolic engineering.

## Methods

### Bacterial strains, media, and reagents

*Escherichia coli* DH5α was used as a host strain for cloning and *n*-butanol production. Strains MG1655 and BW25113 were also used as *n*-butanol production hosts. *Escherichia coli* cells were grown in Luria–Bertani (LB) medium (5 g/L yeast extract, 10 g/L tryptone, and 5 g/L NaCl). For byproduct analysis, TB-glucose medium consisting of TB medium (12 g/L enzymatic casein digest, 24 g/L yeast extract, 9.4 g/L K_2_HPO_4_, and 2.2 g/L KH_2_PO_4_) containing 2% (w/v) glucose was used, and TB-glycerol medium, which is TB medium containing 2% (w/v) glycerol, was used for *n*-butanol production. Ampicillin (100 μg/mL), kanamycin (25 μg/mL), or chloramphenicol (34 μg/mL) was added to media for plasmid selection and maintenance. High-fidelity KOD-Plus-Neo polymerase (Toyobo, Osaka, Japan) was used for PCR. All restriction enzymes, T4 DNA ligase, T4 polynucleotide kinase, and Gibson assembly master mix were purchased from New England Biolabs (Ipswich, MA, USA), and experiments were conducted according to manufacturer instructions.

### Plasmid construction

Primers, plasmids, and sgRNAs used in this study are summarized in Additional file 1: Tables S1, S2, and S3, respectively. For CRISPRi plasmid construction, we used an inverse PCR method for changing the sgRNA sequences [49]. Briefly, we amplified entire regions of the pSECRi plasmid using CRI(pta)-F and CRI(pta)-R primer pairs, followed by ligation of the amplified fragments using T4 DNA ligase and T4 polynucleotide kinase, resulting in the pSECRi-P plasmid. The pSECRi-F, pSECRi-L, pSECRi-A plasmids were constructed using similar methods. For multiplex repression, the pSECRi-P plasmid was digested with *Nco*I/*Xma*I, and the fragment containing sgRNA(P) was gel purified (Promega, Madison, WI, USA). The other fragment containing sgRNA(F) was gel purified by *Nco*I/*Age*I digestion from the pSECRi-F plasmid, followed by ligation of the two purified fragments, resulting in the pSECRi-PF plasmid. Other multiplex CRISPRi plasmids were constructed using a similar method. For pBBR1CRi-PFLA plasmid construction, the RK2 origin of pSECRi-PFLA was removed by *Asc*I/*Fse*I restriction digest, and the remaining fragment was ligated along with the pBBR1 origin of replication fragment prepared using a pSEVA131 plasmid digested with *Asc*I/*Fse*I.

For reporter-plasmid construction, we amplified entire regions of the pREGFP3 plasmid using the pMW(pta)-F and pMW(pta)-R primer pairs, followed by ligation of the amplified fragments using T4 DNA ligase and T4 polynucleotide kinase (New England Biolabs), resulting in plasmid pREGFP3-P. The pREGFP3-F, pREGFP3-L, and pREGFP3-A plasmids were constructed using similar methods.

To change the antibiotic resistance gene in the *n*-butanol-production plasmid, we amplified the entire target region, except for that harboring the *cat* gene, from the pACBB-eGFP plasmid using the AV-F and AV-R primer pairs, and amplified the *bla* gene from the pSEVA131 plasmid using the AI-F and AI-R primer pairs. We assembled the two amplified fragments using the Gibson assembly method [50], resulting in the pACBBA-eGFP plasmid. To incorporate *n*-butanol-pathway enzymes, we amplified the backbone region of the pACBBA-eGFP plasmid using the ACBBA-F and ACBBA-R primers, and *n*-butanol-pathway genes were obtained by *Spe*I/*Xba*I restriction digest from the pAB-HCTA plasmid. The two fragments were then assembled with using the Gibson assembly method [50], resulting in the pABA-HCTA plasmid.

### Gene-reporter assay for CRISPRi activity

*Escherichia coli* cells harboring a reporter plasmid and CRISPRi plasmid were grown in LB medium containing the appropriate antibiotics at 37 °C with shaking at 200 rpm for 8 h. For dCas9 protein induction, 4 mM l-rhamnose was also added to the culture medium. The culture broth was washed once with phosphate-buffered saline (PBS) and resuspended with PBS. Fluorescence and optical density at 600 nm measurements were conducted with the Victor X multi-label plate reader (PerkinElmer, Waltham, MA, USA) using black-walled 96-well polystyrene plates.

### Determination of endogenous mRNA-expression levels

*Escherichia coli* cells harboring CRISPRi plasmid were grown in LB medium containing the appropriate antibiotics at 37 °C with shaking at 200 rpm for 8 h. For dCas9 protein induction, 4 mM l-rhamnose was also added to the culture medium. Total RNA from cultured cells was isolated using the RNeasy protect bacteria mini kit according to manufacturer instructions (Qiagen, Valencia, CA, USA), and cDNA was synthesized from isolated RNA using the ReverTra Ace qPCR RT master mix with gDNA remover (Toyobo, Osaka, Japan). Real-time PCR for mRNA quantification was performed using the CFX Connect real-time PCR detection system (Bio-Rad, Hercules, CA, USA) with iQ SYBR Green supermix (Bio-Rad). The primers used for real-time PCR analysis are listed in Additional file 1: Table S1. Relative gene expression was quantified using 16S ribosomal RNA (rRNA) as an internal control according to the 2^−∆∆Ct^ method [51].

### Quantification of *n*-butanol and other metabolites

For *n*-butanol quantification, the 50-µL headspace of the sealed bottle in which *E. coli* was cultured was used for direct injection for gas chromatography (GC) analysis using a flame ionization detector (FID) attached to an HP-5 column (30 m × 0.320 mm × 0.25 µm) at a flow rate of 1 mL/min. The starting temperature of the oven was set to 40 °C for 3 min, followed by increases at 10 °C/min to 100 °C, maintenance at 100 °C for 3 min, increases at 30 °C/min to 200 °C, and maintenance at 200 °C for 1 min.

For ester-derivative identification, the 50-µL headspace of the sealed bottle in which *E. coli* was cultured was analyzed by GC using an HP-5MS column (30 m × 0.250 mm × 0.25 µm) and a single quadrupole mass spectrometer (Agilent Technologies, Santa Clara, CA, USA). Samples were analyzed at a starting temperature of 40 °C for 3 min, followed by increases at 10 °C/min to 100 °C, maintenance at 100 °C for 3 min, increases at 30 °C/min to 200 °C, and maintenance at 200 °C for 1 min. The ion-source temperature was set to 250 °C, and mass spectra were collected at m/z 41 and m/z 56, with a 1 min solvent delay. The peaks were analyzed using Agilent ChemStation software (Agilent Technologies).

For *n*-butanol and byproduct quantification in medium, 1 mL of cultured cell was centrifuged for 10 min at 12,000 rpm. The supernatant was filtered through a 0.45-µm filter (Millipore, Billerica, MA, USA), and the filtrate was analyzed by GC for *n*-butanol quantification or by high-performance liquid chromatography (HPLC) for byproduct analysis. For *n*-butanol quantification, the filtrate was injected into a gas chromatograph equipped with a FID and a DB-WAX capillary column (30 m × 0.320 mm × 0.5 µm; Agilent Technologies). The oven temperature was initially heated to 60 °C and held for 4 min, followed by increases to 120 °C at a gradient of 15 °C/min, to 230 °C at a gradient of 50 °C/min, and maintenance at 230 °C for 2 min. The injector and detector were maintained at 250 and 300 °C, respectively.

For glycerol and byproduct analyses, the filtrate was injected into an HPLC column equipped with a reflective index detector (Agilent Technologies). Analyses were conducted using an Aminex HPX-87H column (300 × 7.8 mm; Bio-Rad) with the mobile phase (4 mM H_2_SO_4_) injected at 0.5 mL/min and at a temperature of 50 °C. Pure *n*-butanol, acetate, succinate, lactate, and ethanol were purchased from Sigma-Aldrich (St. Louis, MO, USA) and used as external standards for quantification.

### *n*-Butanol fermentation in a bioreactor

*n*-Butanol fermentation was performed in a 1-L stirred-tank fermenter (CNS, Seoul, South Korea) using a working volume of 0.4 L in TB medium containing 2% (w/v) glycerol and 4 mM l-rhamnose with appropriate antibiotics. The fermenter was inoculated with 1% (v/v) of cultured cells, which were grown in LB medium containing 4 mM l-rhamnose with appropriate antibiotics at 37 °C and shaking at 200 rpm overnight. After inoculation, stirrer speed was maintained 250 rpm, and 1 volume of air per volume of liquid per minute (vvm) was air-bubbled through the fermenter. After 6 h, 1 vvm air gas was turned off, and all gas tubes were sealed with pinch clamps to initiate micro-aerobic conditions. The pH was controlled at 7.0 at all times by automated addition of 2 N NaOH solution.

## Additional file

**Additional file 1.** Additional figures and tables.

## Abbreviations

CRISPRi: clustered regularly interspaced short palindromic repeat interference
NADH: reduced β-nicotinamide adenine dinucleotide isopentenyl diphosphate
NAD^+^: β-nicotinamide adenine dinucleotide isopentenyl diphosphate
MAGE: multiplex automated genomic engineering
sRNA: synthetic small-regulatory RNA
sgRNA: single-guide RNA
dCas9: nuclease-deficient Cas9
MVA: mevalonate
IPTG: isopropyl β-d-1-thiogalactopyranoside
CDS: coding DNA sequence
PAM: protospacer adjacent motif
GFP: green fluorescent protein
PCR: polymerase chain reaction
CAT: chloramphenicol acetyltransferase
TB: terrific broth
LB: Luria–Bertani
PBS: phosphate-buffered saline
qPCR: quantitative PCR
mRNA: messenger RNA
rRNA: ribosomal RNA
vvm: volume of air per volume of medium per minute
GC: gas chromatography
FID: flame ionization detector
HPLC: high-performance liquid chromatography
