# Supplementary material for: CRISPR interference-guided multiplex repression of endogenous competing pathway genes for redirecting metabolic flux in Escherichia coli
Source: Microb Cell Fact. 2017 Nov 3;16:188. doi: 10.1186/s12934-017-0802-x (PMC5670510; doi:10.1186/s12934-017-0802-x)
Supplement: Supplementary file 1 — Additional file 1. Additional figures and tables. [file 12934_2017_802_MOESM1_ESM.doc]

**Additional file**

**CRISPR interference-guided multiplex repression of endogenous competing pathway genes for redirecting metabolic flux in *Escherichia coli***

Seong Keun Kim^1†^, Wonjae Seong^1,2†^, Gui Hwan Han^1^, Dae-Hee Lee^1,2^,*, and Seung-Goo Lee^1,2^,*

^1^ Synthetic Biology and Bioengineering Research Center, Korea Research Institute of Bioscience and Biotechnology (KRIBB), Daejeon 34141, Republic of Korea

^2^ Department of Biosystems and Bioengineering, KRIBB School of Biotechnology, University of Science and Technology (UST), Daejeon 34113, Republic of Korea

^†^These authors contributed equally to this work.

* Corresponding authors:

Dae-Hee Lee, Ph.D.

Synthetic Biology and Bioengineering Research Center, Korea Research Institute of Bioscience and Biotechnology (KRIBB), Daejeon 34141, Republic of Korea

Tel: +82 42 879 8225; Fax: +82 42 860 4489; E-mail: dhlee@kribb.re.kr

Seung-Goo Lee, Ph.D.

Synthetic Biology and Bioengineering Research Center, Korea Research Institute of Bioscience and Biotechnology (KRIBB), Daejeon 34141, Republic of Korea

Tel: +82 42 860 4373; Fax: +82 42 860 4489; E-mail: sglee@kribb.re.kr

E-mail addresses: SKK: draman97@kribb.re.kr; WS: winise@kribb.re.kr; GHH: ghhan@kribb.re.kr; DL: dhlee@kribb.re.kr; SL: sglee@kribb.re.kr

**Table S1**. Primers used in this study.

| Primers | Sequences of oligonucleotides (5ʹ to 3ʹ) | Remarks |
| --- | --- | --- |
| For CRISPRi plasmids construction | |  |
| CRI(pta)-F | tattatgctgactagtattatacctaggac | Repression for *pta* |
| CRI(pta)-R | atacgggacagttttagagctagaaatagc |
| CRI(pta2)-F | actagtattatacctaggac | Repression for *pta* |
| CRI(pta2)-R | tttcgttaccgccgatttgggttttagagctagaaatagc |
| CRI(pta3)-F | actagtattatacctaggac | Repression for *pta* |
| CRI(pta3)-R | gatcacgccaaggctgacgcgttttagagctagaaatagc |
| CRI(pta4)-F | actagtattatacctaggac | Repression for *pta* |
| CRI(pta4)-R | ccggtacgcggctgagcgatgttttagagctagaaatagc |
| CRI(frdA)-F | tcttgccattactagtattatacctaggac | Repression for *frdA* |
| CRI(frdA)-R | tcggcttgaagttttagagctagaaatagc |
| CRI(ldhA)-F | caaaacagtacactagtattatacctaggac | Repression for *ldhA* |
| CRI(ldhA)-R | tgctataaagttttagagctagaaatagc |
| CRI(adhE)-F | tgccagtttcactagtattatacctaggac | Repression for *adhE* |
| CRI(adhE)-R | tattcacgctgttttagagctagaaatagc |
|  | |  |
| For reporter plasmid construction | |  |
| pMW(pta)-F | tattatgctgaataattttgtttaactttaaga | pREGFP3-P construction |
| pMW(pta)-R | atacgggacacggtctagagggaaaccgttgtg |  |
| pMW(frdA)-F | tcttgccattaataattttgtttaactttaaga | pREGFP3-F construction |
| pMW(frdA)-R | tcggcttgaaaggtctagagggaaaccgttgtg |
| pMW(ldhA)-F | caaaacagtacaataattttgtttaactttaaga | pREGFP3-L construction |
| pMW(ldhA)-R | tgctataaacggtctagagggaaaccgttgtg |
| pMW(adhE)-F | tgccagtttcaataattttgtttaactttaaga | pREGFP3-A construction |
| pMW(adhE)-R | tattcacgctgggtctagagggaaaccgttgtg |
|  |  |  |
| For n-butanol production plasmid construction | |  |
| AV-F | ataacttcaggacctgcagcggtgatgctgccaacttact | pACBBA-eGFP construction |
| AV-R | ggagtccaagactagtacatgcggtgtgaaatac |
| AI-F | atgtactagtcttggactcctgttgataga |
| AI-R | gctgcaggtcctgaagttatgaaaaggacaagggtcgtcc |
| ACBBA-F | ggtatttcacaccgcatgtactagtcttggactcctgttg | pABA-HCTA construction |
| ACBBA-R | tgcccgctttccagtcgggtctagataagttcaggatgaa |

| For qRT-PCR | |  |
| --- | --- | --- |
| RT-16S-F | cgtgtatgaagaaggccttcg | 16S rRNA |
| RT-16S-R | ctgagcgtcagtcttcgtcc |
| RT-pta-F | atgtgctgatggaagagatcg | *pta* mRNA |
| RT-pta-R | gccctgagacataacgaagac |
| RT-frdA-F | caaccgaaatgacccaactg | *frdA* mRNA |
| RT-frdA-R | tcaaccagaatatccagcacg |
| RT-ldhA-F | acgagtcctttggctttgag | *ldhA* mRNA |
| RT-ldhA-R | gatcataggctggaacacgg |
| RT-adhE-F | actaacccgacttcaactgc | *adhE* mRNA |
| RT-adhE-R | catcagtgcgttagacagttc |

**Table S2**. Plasmids used in this study.

| Name | Description | Refs |
| --- | --- | --- |
| CRISPRi Plasmids |  |  |
| pSEVA221 | RK2 ori, Kan^R^ | [19, 44] |
| pSECRi | P*_rhaBAD_::cas9*(D10A, H840A) and constitutive sgRNA expression cassette in pSEVA221 | [16] |
| pSECRi-P | pSECRi expressing sgRNA(P) | This study |
| pSECRi-P2 | pSECRi expressing sgRNA(P2) | This study |
| pSECRi-P3 | pSECRi expressing sgRNA(P3) | This study |
| pSECRi-P4 | pSECRi expressing sgRNA(P4) | This study |
| pSECRi-F | pSECRi expressing sgRNA(F) | This study |
| pSECRi-L | pSECRi expressing sgRNA(L) | This study |
| pSECRi-A | pSECRi expressing sgRNA(A) | This study |
| pSECRi-PF | pSECRi expressing sgRNA(P), sgRNA(F) | This study |
| pSECRi-PL | pSECRi expressing sgRNA(P), sgRNA(L) | This study |
| pSECRi-PA | pSECRi expressing sgRNA(P), sgRNA(A) | This study |
| pSECRi-FL | pSECRi expressing sgRNA(F), sgRNA(L) | This study |
| pSECRi-FA | pSECRi expressing sgRNA(F), sgRNA(A) | This study |
| pSECRi-LA | pSECRi expressing sgRNA(L), sgRNA(A) | This study |
| pSECRi-PFL | pSECRi expressing sgRNA(P), sgRNA(F), sgRNA(L) | This study |
| pSECRi-PFA | pSECRi expressing sgRNA(P), sgRNA(F), sgRNA(A) | This study |
| pSECRi-PLA | pSECRi expressing sgRNA(P), sgRNA(L), sgRNA(A) | This study |
| pSECRi-FLA | pSECRi expressing sgRNA(F), sgRNA(L), sgRNA(A) | This study |
| pSECRi-PFLA | pSECRi expressing sgRNA(P), sgRNA(F), sgRNA(L), sgRNA(A) | This study |
| pBBR1CRi-PFLA | P*_rhaBAD_::cas9*(D10A, H840A) and sgRNA(P), sgRNA(F), sgRNA(L), sgRNA(A) cassettes in pSEVA231 | This study |
|  |  |  |
| Reporter Plasmids |  |  |
| pREGFP3 | pJ23100-*gfp* in pMW7 | [16] |
| pREGFP3-P | pREGFP3 containing sgRNA(P) binding site | This study |
| pREGFP3-F | pREGFP3 containing sgRNA(F) binding site | This study |
| pREGFP3-L | pREGFP3 containing sgRNA(L) binding site | This study |
| pREGFP3-A | pREGFP3 containing sgRNA(A) binding site | This study |
|  |  |  |
| Production Plasmids |  |  |
| pSEVA131 | pBBR1 origin, Amp^R^ | [44] |
| pACBB-eGFP | *lacP′:: egfp,* p15A origin, Cam^R^ | [45] |
| pACBBA-eGFP | *lacP′:: egfp,* p15A origin, Amp^R^ | This study |
| pAB-HCTA | *lacP′:: atoB*, *lacP′:: hbd*, *lacP′:: crt, lacP′:: ter, lacP′:: adhE2,* p15A origin, Cam^R^ | Unpublished |
| pABA-HCTA | *lacP′:: atoB*, *lacP′:: hbd*, *lacP′:: crt, lacP′:: ter, lacP′:: adhE2,* p15A origin, Amp^R^ | This study |
|  |  |  |

**Table S3**. sgRNA binding sites used in this study.

| sgRNA name | Target gene | Binding site with PAM domain (5ʹ to 3ʹ) |
| --- | --- | --- |
| sgRNA(P) | *pta* | cagcataataatacgggaca**CGG** |
| sgRNA(P2) | *pta* | tttcgttaccgccgatttgg**CGG** |
| sgRNA(P3) | *pta* | gatcacgccaaggctgacgc**TGG** |
| sgRNA(P4) | *pta* | ccggtacgcggctgagcgat**AGG** |
| sgRNA(F) | *frdA* | aatggcaagatcggcttgaa**AGG** |
| sgRNA(L) | *ldhA* | gtactgttttgtgctataaa**CGG** |
| sgRNA(A) | *adhE* | gaaactggcatattcacgct**GGG** |


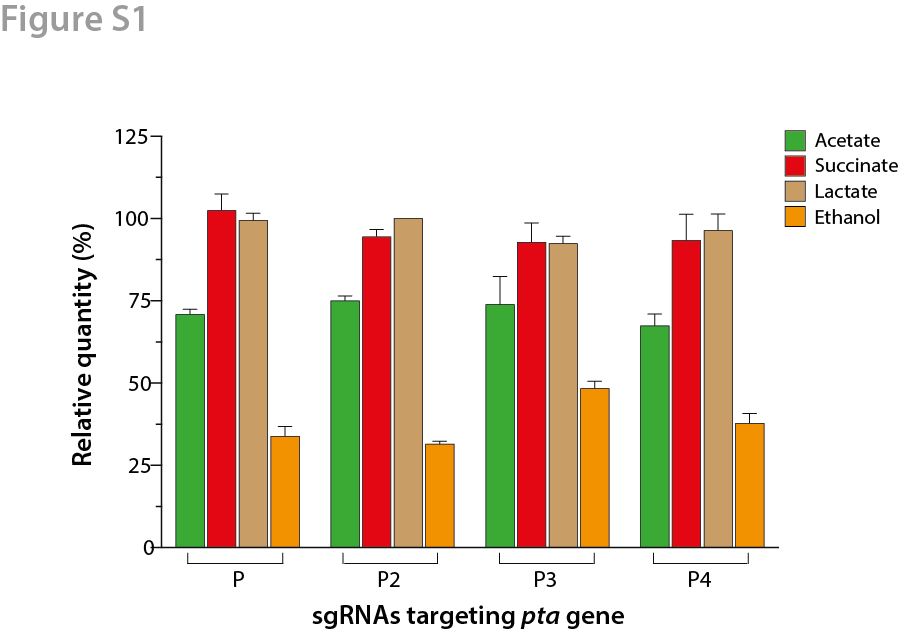


**Figure S1.** Comparison of the effect of CRISPRi-mediated *pta* repression using different sgRNAs on byproduct formation in *E. coli*. P2 sgRNA repressed the *pta* gene by targeting the ribosome-binding site, and P3 and P4 sgRNAs are targeting the CDS of *pta* gene at different locations. *E. coli* cells transformed with individual CRISPRi plasmid for *pta* repression were grown in TB-glucose medium containing 4 mM L-rhamnose at 37°C for 36 h. Byproducts were quantified by HPLC. Data represent averages from two biological cultures, and error bars show the standard deviation (SD).


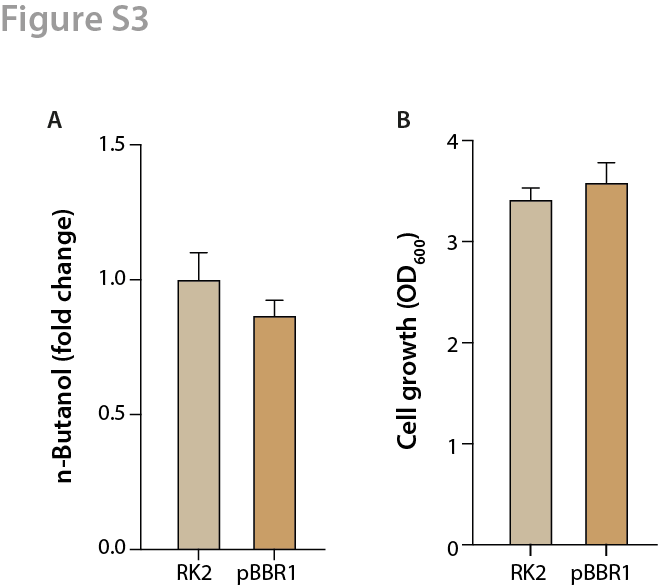


**Figure S2**. Comparison of the effect of CRISPRi plasmid copy number on *n*-butanol production in *E. coli*. To increase the production of the dCas9-sgRNA complex, the origin of replication in the pSECRi plasmid was switched from RK2 (low copy number) to pBBR1 (medium copy number). *E. coli* cells transformed with individual CRISPRi plasmid repressing all four genes (*pta*, *frdA*, *ldhA*, and *adhE*) were grown in TB-glycerol medium containing 4 mM L-rhamnose at 37°C for 48 h. *n*-Butanol levels were determined by GC. The fold change of *n*-butanol was calculated by dividing the *n*-butanol content produced from the tested cells by the *n*-butanol concentration yielded from a control containing the pSECRi with RK2 origin. Data represent the averages of three biological cultures, and error bars show the standard deviation (SD).


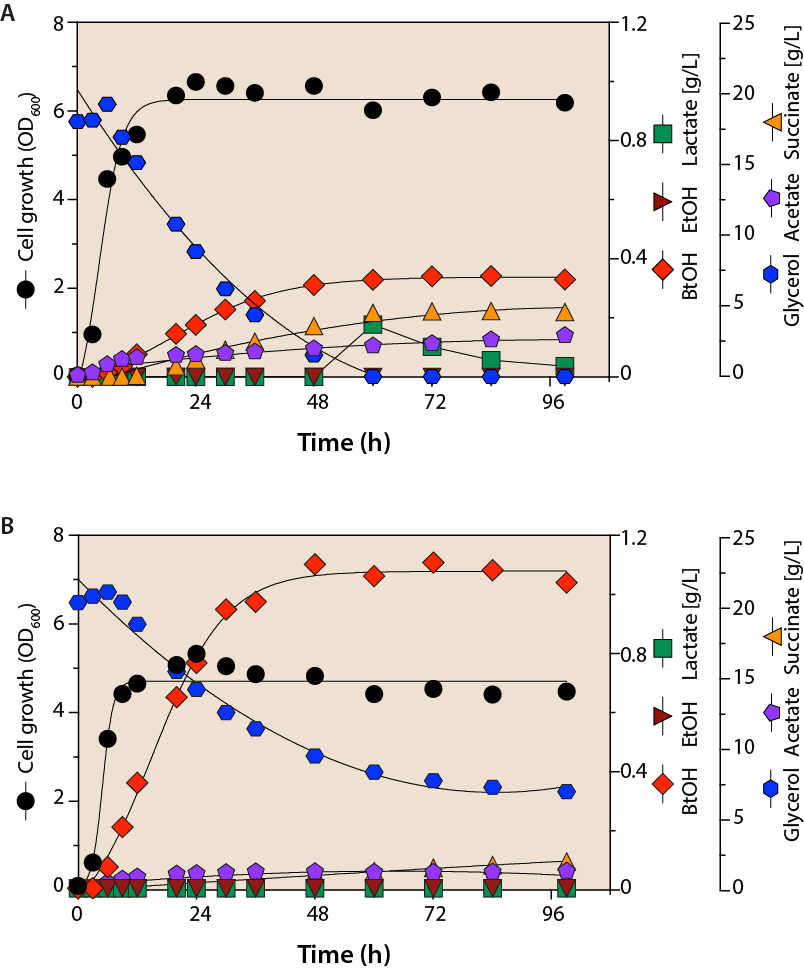


**Figure. S3** Batch-fermentation of *E. coli*. The BW25113 strain containing (a) the pABA-HCTA and pSEVA221 plasmids or (b) the pABA-HCTA and pSECRi-PFLA plasmids was grown in TB containing 20 g/L glycerol and 4 mM L-rhamnose at 37°C. After a 6 h fermentation, 1 vvm of air was reduced to 0 vvm to initiate micro-aerobic conditions. Concentrations of *n*-butanol and byproducts were measured by GC and HPLC, respectively.
